# The Phosphocarrier Protein HPr Contributes to Meningococcal Survival during Infection

**DOI:** 10.1371/journal.pone.0162434

**Published:** 2016-09-21

**Authors:** Ana Antunes, Meriem Derkaoui, Aude Terrade, Mélanie Denizon, Ala-Eddine Deghmane, Josef Deutscher, Isabel Delany, Muhamed-Kheir Taha

**Affiliations:** 1 Institut Pasteur, Unité des Infections Bactériennes Invasives, Paris, France, 75724 Paris Cedex 15, France; 2 Micalis Institute, INRA, AgroParisTech, Université Paris-Saclay, 78350, Jouy-en-Josas, France; 3 Centre National de la Recherche Scientifique, UMR8261 (affiliated with Univ. Paris Diderot, Sorbonne Paris Cité), Expression Génétique Microbienne, Institut de Biologie Physico-Chimique, 75005, Paris, France; 4 Novartis Vaccines and Diagnostics s.r.l. (a GSK company), Via Fiorentina 1, 53100, Siena, Italy; University of Würzburg, GERMANY

## Abstract

*Neisseria meningitidis* is an exclusively human pathogen frequently carried asymptomatically in the nasopharynx but it can also provoke invasive infections such as meningitis and septicemia. *N*. *meningitidis* uses a limited range of carbon sources during infection, such as glucose, that is usually transported into bacteria via the phosphoenolpyruvate (PEP):sugar phosphotransferase system (PTS), in which the phosphocarrier protein HPr (encoded by the *ptsH* gene) plays a central role. Although *N*. *meningitidis* possesses an incomplete PTS, HPr was found to be required for its virulence. We explored the role of HPr using bioluminescent wild-type and Δ*ptsH* strains in experimental infection in transgenic mice expressing the human transferrin. The wild-type MC58 strain was recovered at higher levels from the peritoneal cavity and particularly from blood compared to the Δ*ptsH* strain. The Δ*ptsH* strain provoked lower levels of septicemia in mice and was more susceptible to complement-mediated killing than the wild-type strain. We tested whether meningococcal structures impacted complement resistance and observed that only the capsule level was decreased in the Δ*ptsH* mutant. We therefore compared the transcriptomic profiles of wild-type and Δ*ptsH* strains and identified 49 differentially expressed genes. The HPr regulon contains mainly hypothetical proteins (43%) and several membrane-associated proteins that could play a role during host interaction. Some other genes of the HPr regulon are involved in stress response. Indeed, the Δ*ptsH* strain showed increased susceptibility to environmental stress conditions. Our data suggest that HPr plays a pleiotropic role in host-bacteria interactions most likely through the innate immune response that may be responsible for the enhanced clearance of the Δ*ptsH* strain from blood.

## Introduction

*Neisseria meningitidis* is a Gram-negative bacterium belonging to the β-subgroup of proteobacteria. This bacterium is a facultative commensal and its known reservoir is exclusively human. *N*. *meningitidis* colonizes the nasopharynx of about 10% of the general population [1]. Direct person-to-person spread of meningococci occurs by droplet transmission [2, 3]. Upon acquisition of hyper-invasive isolates, *N*. *meningitidis* may cross the epithelial barrier to enter the bloodstream where it can replicate causing septicemia and/or cross the blood-brain barrier causing meningitis, with a global fatality rate of 10% [4]. For invasive infection, the ability to evade killing by complement is of paramount importance for *N*. *meningitidis* [5]. Meningococcus has evolved several redundant mechanisms to evade the host innate responses at the site of colonization and during systemic growth. These include the capsule which is important for serum resistance [6] and essential to sustain septicemia in a transgenic mouse model expressing human transferrin [7], the factor H binding protein (fHbp) [8, 9], neisserial surface protein A (NspA) [10, 11] and PorB2 [12], that regulate the alternative pathway (AP) of complement activation, lipooligosaccharide (LOS) sialic acid, which inhibits complement deposition, and NalP that cleaves human complement C3 [13]. During invasive infection, meningococcal growth requires iron sources such as the human transferrin (hTf) and carbon sources [4]. Nevertheless, *N*. *meningitidis* is able to grow on minimal media utilizing only a limited range of carbon sources including glucose, maltose, lactate, pyruvate and some amino acids such as glutamate [14–16]. It catabolizes glucose primarily via the Entner-Douderoff (ED) and to a lesser extent via the pentose phosphate (PP) pathway [17]. The upper part of the Embden-Meyerhof-Parnas (EMP) glycolytic pathway does not contribute to pyruvate synthesis due to the lack of the phosphofructokinase gene in meningococcus [15]. Previous studies have shown that different metabolic pathways, such as lactate metabolism, the glutathione metabolism or the denitrification pathway, play a major role in meningococcal pathogenesis, therefore showing that meningococcal metabolism is intimately linked to pathogenesis (for a review see [18]). In an infant rat experimental model of infection, about half of the genes found to be essential during bacteremia infection by *N*. *meningitidis* have a metabolism-related function [19], including Enzyme I (EI) and the histidine-containing phosphocarrier HPr, two components of the phosphoenolpyruvate (PEP): sugar phosphotransferase system (PTS) that are involved in the regulation of sugar transport according to carbon source availability. Besides EI and HPr, that are present in the cytoplasm, the PTS is also composed of the carbohydrate-specific transporter enzyme IIC (EIIC), which is located in the membrane, and EIIA and EIIB, which can be fused to the N- or C-terminus of the EIIC or be distinct cytoplasmic proteins [20].

*N*. *meningitidis* MC58 possesses the general enzymes of the PTS system, the EI (NMB2044) as well as HPr (NMB2045) and two EIIA components, one of the mannose and the other of the fructose PTS family (NMB2046 and NMB0736, respectively). However, the PTS of meningococcus is incomplete because it lacks both the B and C domains necessary for the uptake of carbohydrates by this system. HPr not only plays a role in the transfer of the phosphate group that allows the phosphorylation of the sugar bound to the EIIC and its subsequent entry into the cytoplasm, but it is also implicated in the regulation of expression of several gene targets such as virulence factors (see review [21]). In this work we explored the role of HPr during infection using transgenic mice expressing the human transferrin that allows meningococcal growth by providing both iron and carbon sources [22].

## Materials and Methods

### Ethics statement

This study was carried out in strict accordance with the European Union Directive 2010/63/EU (and its revision 86/609/EEC) on the protection of animals used for scientific purposes. Our laboratory has the administrative authorization for animal experimentation (Permit Number 75–1554) and the protocol was approved by the Institut Pasteur Review Board that is part of in the Regional Committee of Ethics of Animal Experiments of the Paris region (CETEA 2013–0190). All the invasive procedures were performed under anesthesia and all possible efforts were made to minimize animal suffering. The animals were euthanatized by injection of high dose of chemical anesthetics (pentobarbital) which was performed before blood sampling.

Normal human serum (NHS) was obtained from healthy adult volunteers, who, at the time, had not received any meningococcal vaccines, and stored (as individual sera) at -80°C until used. A pool of 9 different human sera was prepared just before use. Sera were obtained anonymously from the (Centre of the plate-forme Investigation Clinique et Accès aux Ressources Biologiques, ICAReB, Institut Pasteur) who obtained written informed consent from donors. The clinical research protocols, constitution of collection of human biological samples and informed consent received all permits from local, regional and national ethics committees.

Mice sera were obtained from adult BALB/c non-infected mice.

### Bacterial strains and growth conditions

*N*. *meningitidis* strains were routinely cultured on GCB agar plates supplemented with Kellogg supplements [23] at 37°C in a 5% CO_2_-95% air atmosphere at 95% humidity. Strains were stored at −80°C in GC medium with 15% glycerol. When required, kanamycin (Kan), spectinomycin (Spc) and erythromycin (Ery) were added at 100 μg/ml, 75 μg/ml and 5 μg/ml respectively. The bioluminescent strain MC58 LUX is a derivative of the MC58 strain (serogroup B belonging to the cc32) [24], that was transformed of MC58 by chromosomal integration of the operon coding for the luciferase (*luxCDABE*) under the control of the promoter of *porB* meningococcal gene as previously described [25]. *Escherichia coli* DH5α [26] strains were grown on LB medium [27] at 37°C. Phenotypic and genotypic characteristics of all *N*. *meningitidis* strains used in this study are listed in Table 1.

**Table 1:** Strains used in this study.

Strain	Features	Origin
MC58	*Nm* laboratory-adapted reference strain, sequenced strain	Ref [24]
MC58 ΔptsH	*ptsH* (NMB2045) deletion mutant of MC58, kan^R^	This study
MC58 ΔptsH-C	MC58 *ΔptsH* complemented with *ptsH*, kan^R^, Spec^R^	This study
MC58 lux	MC58 strain, in wich PporB-luxCDABE-aph3’ inserted into pilE chromosome	This study
MC58 lux ΔptsH	MC58 lux with *ptsH* (NMB2045) deletion mutant, kan^R^, Erm^R^	This study
MC58 lux ΔptsH-C	MC58 lux ΔptsH complemented with *ptsH*, kan^R^, Erm^R^, Spec^R^	This study
MC58 ΔnspA	nspA (NMB0663) deletion mutant of MC58, Kan^R^	Ref [50]
MC58 ΔnalP	nalP (NMB1969) deletion mutant of MC58, Kan^R^	Ref [51]

### Construction of *ptsH* knockout and complemented strains

DNA manipulations were carried out routinely as described for standard laboratory methods [28]. Genomic and plasmid DNA were extracted using the Qiamp DNA mini kit (Qiagen, Courtaboeuf, France) and the PureLink^™^ Quick plasmid DNA Miniprep Kit (Life Technologies, Saint Aubin, France), according to manufacturer’s instructions. All restriction and modification enzymes were used according to manufacturer’s recommendations. All primers used in this study were purchased from Sigma and are listed in Table 2.

**Table 2:** Primers used in this study.

Name	Sequence (5’-3’)*	Features
ptsM1-F	5'-CATCACACACGAAACCATAGG-3'	Checking *ptsH* KO
ptsI3-R	5'-CCTTGAAGTCGGCTTCTTCC-3'	
ptsHRT-F3	5'-ATGGGGCTGATGATGCTCGC-3'	Amplification of a 111bp fragment of *ptsH* gene by qRT-PCR
ptsHRT-R3	5'-GTAGCCGTTGATTAAGTCGG-3'	
rpoBRT-F	5'-GGCAGCGGTAAGAAAGAAGA-3'	Amplification of a 103bp fragment of *rpoB* gene by qRT-PCR
rpoBRT-R	5'-CGAATACAGGAGAGGCGAAA-3'	
aad3-XbaI	5’ -ATTCAG*TCTAGA*GCTTAGTGCATCTAACGCTTG- 3’	Amplification of the spectinomycin cassete to replace *cat* gene into pCOMP_RBS_
aad1-KpnI-2	5’-ATTCAG*GGATCC*AAGCTCTCGGGTAACATCAAG- 3’	
ptsH-F-NdeI	5'-attcagcatATGCTCAAACAATCCATCGAAATCATC-3'	Amplification of *ptsH* gene to insert in pCOMP_RBS_-spec
ptsH-R-NsiI	5'-attcagatgcatTTATTCGCCCTCGCCGAAGTAGCCGTTG-3'	
ptsHKOKan-NcoI-F	5'-cggcgtctgctgccATGgggaagcttccagcg-3'	Site-directed mutagenesis to insert a NcoI site at the begin of kan cassette on the pBluescript-ΔptsH/aphA3
ptsHKOKan-NcoI-R	5'-cgctggaagcttcccCATggcagcagacgccg-3'	
ptsHKOKan-ApaI-F	5'-ctggattgttttagGGcccatcaacggctacttcg-3'	Site-directed mutagenesis to insert a ApaI site at the end of kan cassette on the pBluescript-ΔptsH/aphA3
ptsHKOKan-ApaI-R	5'-cgaagtagccgttgatgggCCctaaaacaatccag-3'	
Eram1-NcoI	5'-attcagccatggGCAAACTTAAGAGTGTGTTGA-3'	Amplification of erythromycin resistance cassette *erm*
Eram3-ApaI	5'-attcaggggcccAAGCTTGCCGTCTGAATGGGACCTCTTTTAGCTTCTTGG-3'	
COMCFW-NVD	5'-cctcgagccgctgaccgaagg-3'	Checking complementation on the intergenic region NMB1428-NMB1429.
COMCREV-NVD	5'-accggcatcggcaactacac-3'	

*Underlined letters indicate restriction enzyme sites.

In order to construct a *ptsH* null mutant the MC58 strain was transformed with a linearized plasmid pBluescript-ΔptsH/aphA3, which contains a kanamycin cassette flanked by the upstream and downstream regions of *ptsH*. Transformants were selected on GCB medium with kanamycin and the correct double homologous recombination event resulting in knockout of the *ptsH* gene was verified by PCR with appropriate verification primers (Table 2). In addition, the absence of HPr was confirmed by qRT-PCR (data not shown). In order to introduce the *ptsH* mutation in the MC58 Lux strain (that was already kanamycin resistance), the plasmid pBluescript-ΔptsH/aphA3 was modified by creating unique restriction sites for the enzymes NcoI and ApaI at the beginning and end of the *aphA3* gene, respectively using the QuickChange II XL Site-Directed Mutagenesis kit (Agilent Technologies) following the manufacturer’s instructions. The *aphA3* gene was replaced by the *erm* cassette amplified by using primers Eram1-NcoI and Eram3-ApaI (Table 2). Transformants were selected on GCB medium with erythromycin and the correct double homologous recombination event resulting in knockout of the *ptsH* gene was verified by PCR with appropriate verification primers (Table 2).

For the complementation of the MC58 Δ*ptsH* null mutant or the MC58 LUX Δ*ptsH*, the *ptsH* gene under the control of the constitutive P_tac_- promoter was re-inserted into the intergenic region between the converging open reading frames (ORFs) NMB1428 and NMB1429. The two Δ*ptsH* strains were transformed with pComP_RBS_ptsH, a pComP_RBS_-derived plasmid [9], in which the *cat* gene encoding the chloramphenicol acetyl transferase was replaced with a spectinomycin cassette that had been amplified by using primers aad1-KpnI-2 and aad3-XbaI. In addition the *ptsH* gene was amplified from the MC58 strain with the primers ptsH-F-NdeI/ptsH-R-NsiI and cloned as a 272 bp NdeI/NsiI fragment downstream of the P_tac_ promoter. Transformants were selected on spectinomycin and the correct insertion by a double homologous recombination event was verified by PCR analysis with appropriate primers (Table 2). The resulting strains were designated MC58 *Δpts*H-C_RBS_ and MC58 LUX *Δpts*H-C_RBS_.

### Mice experimental infection and analysis

We have previously described the use of 7 to 8 weeks-old female BALB/c transgenic mice expressing the human transferrin (hTf) for infection by intra-peritoneal (ip) injection as an experimental model to study meningococcal infection [22]. Mice were in-house bred and were kept in a biosafety containment facility, in filter-topped cages with sterile litter, water and food, according to institutional guidelines.

Mice were challenged with 500 μl of bacterial suspension in PBS standardized at 2X10^7^ CFU/ml. The number of viable bacteria in the initial inoculum was determined by plating serial dilutions on GCB plates. Bacterial counts from blood samples and peritoneum washes were determined at 6 h post-infection, by plating serial dilutions on GCB agar plates with appropriate antibiotics. Results are expressed as percentage of bacterial survival in mice ((viable bacteria at 6 h /initial inoculum) X100). After 18 h incubation at 37°C under a 5% CO_2_ atmosphere, colonies were counted to determine the number of colony forming units (CFU). The results of several experiments (at least three) were combined and the two-tailed Mann-Whitney test was used to determine statistical significances (GraphPad Prism version 6.0a for Mac, GraphPad Software, CA, USA).

In another set of experiments meningococcal infection was followed by dynamic imaging as previously described [25]. Mice were divided in three groups and infected separately with bioluminescent strains of MC58 LUX, *ΔptsH*LUX, and *ΔptsH*-C_RBS_LUX. Quantification was performed using the total photons emitted per second by each mouse after 30 min, 2 h, 6 h, 8 h and 24 h of infection by defining regions of interest, using Living Image 3.1 software (Xenogen Corp.). The results were pooled and analyzed using the two-way ANOVA multiple comparison test to determine statistical differences (GraphPad Prism version 6.0a for Mac, GraphPad Software, CA, USA).

### Flow cytometry of inflammatory cells recruitment

Peritoneum washes taken from infected mice at 6 h post-challenge were harvested by centrifugation at 4 000 x g for 1 min. The pellet was carefully washed and stained for 30 min in the dark with CD3 (clone 17A2, BD Pharmingen), CD19 (clone 1D3, BD Pharmingen), GR1 (clone RB6-8C5, BD Pharmingen) or F4/80 (clone BM8, eBiosciences). After washes, samples were fixed in 0.5 ml of RPMI containing 0.5% formaldehyde and subjected to FACS analysis using a FACSCalibur flow cytometer (BD Biosciences, France). Cells were visualized for a total of 10 000 events per sample and data were subsequently analyzed using FlowJo 8.7 Software.

### Cytokine immunoassays measurement

Immunoreactive IL-6, KC and TNF-α assays were performed both on blood samples and peritoneum washes taken from infected mice at 2 h and 6 h post-infection using ELISA kit (Quantikine, R&D Systems Europe) and analyzed according to the manufacturer’s instructions.

### *In-vitro* survival of bacteria in human and mouse sera

We determined the survival of *N*. *meningitidis* MC58, *ΔptsH* and *ΔptsH*-C_RBS_ strains in pools of mouse and human sera. Bacteria from an overnight culture on GCB agar plates were harvested and a bacterial suspension was prepared in Hanks balanced salt solution (HBSS) at ~ 10^7^ CFU/ml. Approximately 500 CFUs of bacteria were incubated with 25% (v/v) of serum in a final reaction volume of 100 μl. Aliquots of 25 μl were plated in duplicate at the start of the assay (*t*_*0*_) and after incubating the reaction mixture at 37°C for 20, 40, and 60 min (*t*_*60*_). Colony counts were ascertained after incubation overnight, and the percent survival was determined by comparing the viable CFU at *t*_*X*_ compared to that at *t*_*0*_.

### C3b deposition on *N*. *meningitidis* surface

Approximately 10^7^ bacteria/ml in HBSS with 1 mM MglCl_2_+ 1.26 mM CaCl_2_ HBSS (Life Technologies) were incubated with 25% of either mouse sera or human pooled sera in a final volume of 100 μl for 60 or 30 min at 37°C, respectively. Bacteria were then harvested by centrifugation at 4000 x g for 3 min. Samples were stained with mouse C3 (clone 11H9, Hycult Biotech) or human C3 (clone 755, Abcam) for 15 min. After washing, samples were stained with appropriate fluorescein IgG and fixed in HBSS containing 0.5% formaldehyde. Deposited C3b was measured by FACS analysis using FACSCalibur flow cytometer (BD Biosciences, France). Results from at least two experiments were analyzed and a two-tailed Mann-Whitney test was used to determine the statistical significance of observed differences (GraphPad Prism version 6.0a for Mac, GraphPad Software, CA, USA).

### Western blotting and LOS extraction and detection

*N*. *meningitidis* colonies from overnight plate cultures were resuspended in phosphate-buffered saline (PBS 1X) to an optical density of 0.5 at 600 nm (OD_600_). Samples of 1 ml were collected and the pellet resuspended in 100 μl of 2× SDS-PAGE loading buffer (100 mM Tris HCl [pH 6.8], 5% SDS, 0.2% bromophenol blue, 20% glycerol, 10% beta-mercaptoethanol, 100 mM dithiothreitol [DTT]). 10 μl of total extracts were separated on a 4–14% gradient SDS polyacrylamide gel and subsequently transferred onto a nitrocellulose membrane. Membranes were blocked overnight at 4°C by agitation in blocking solution (10% skim milk, 0.05% Tween 20, in PBS) and then incubated for 60 min at 37°C with either anti-Fhbp [29], anti-NspA, anti-PorA, anti-PorB [30] or anti-NalP [13] antibodies. After washing, the membranes were incubated in a 1:5000 dilution of appropriate peroxidase-conjugated secondary IgG antibodies (GE Healthcare Life Sciences) and detected using Pierce ECL Plus Substrate (Thermo Scientific). LOS extraction was performed as previously described [31]. Briefly, meningococcus colonies from overnight cultures were resuspended in buffer A (20 mM Tris HCl, 1 mM MgSO_4_, pH 7.5) at OD_600_ 0.4. An aliquot of 1.5 ml culture suspension was centrifuged and the pellet resuspended in lysis buffer (Tris 1 M pH 6.8, 2% SDS, 4% beta-mercaptoethanol, 10% glycerol, 0.1% bromophenol blue) and incubated at 100°C for 10 min, followed by addition of 25 μg of proteinase K and further incubation at 60°C for 60 min. The lysed sample was then separated on a 14% SDS polyacrylamide gel and silver stained using the Pierce ^®^ Silver Stain kit, according to manufacturer’s instructions.

### Quantification of Capsule production by ELISA

Bacterial suspensions (2.5 x 10^7^ CFU/ml) in PBS of MC58, *ΔptsH* and *ΔptsH*-C_RBS_ were inactivated for 30 min at 56°C and then coated overnight on wells of maxisorp microtiter plates (NUNC) at 45°C. After washing, capsule analysis was performed using anti-serogroup B mouse monoclonal antibodies (National Institute for Biological Standards and Control, UK) 1000-fold diluted. After incubation at 37°C for 1 h the wells were washed and 5000-fold diluted peroxidase-coupled secondary mouse anti-IgG antibodies were added. After washing, colorimetric detection was obtained by adding the substrate o-phenylenediamine and hydrogen peroxide dissolved in 0.1 M citrate buffer, pH 5.5. The plates were kept for 15 min in the dark before adding 50 μl of 2 N sulfuric acid to stop the reaction. The formation of orange color was subsequently measured with a Multiskan Ascent plate reader (MTX Lab Systems) at OD_492_. Relative capsule quantification was calculated for all the three strains by normalizing their results obtained to a non-capsulated strain (MC58 Δ*ctr*A). Results from at least three independent experiments were analyzed with a one-way ANOVA multiple comparison test to determine the statistical significance of observed differences (GraphPad Prism version 6.0a for Mac, GraphPad Software, CA, USA).

### Total RNA preparation and reverse-transcription-polymerase Chain Reaction (RT-PCR)

Bacterial cultures were grown in liquid medium to an OD_600_ of 0.5 and then added to an equal volume of frozen medium to bring the temperature immediately to 4°C. Total RNA was isolated using the RNeasy kit (Qiagen) following the manufacturer’s instructions. Total RNA was extracted from three independent bacterial cultures and 15 μg of each sample were pooled. Three independent pools were prepared for each condition tested. 2 μg of total RNA treated with Turbo DNA-free DNase (Ambion) was reverse transcribed using random hexamer primers and AMV reverse transcriptase (Promega) following the manufacturer’s instructions. Real-time quantitative RT-PCR was performed twice with triplicate biological samples in a 25 μl reaction mixture containing 80 ng of cDNA, 1X Brilliant II SYBR green quantitative PCR master mix (Agilent) and 0.2 μM of gene-specific primers. The primers used in this study are listed in Table 2. Amplification and detection of specific products were performed with an 7300 Real-Time PCR System (Applied Biosystems) using the following procedure: 95°C for 10 min, followed by 40 cycles of 95°C for 30 s, 60°C for 1 min and 72°C for 30 s then ending with a dissociation curve analysis. The *rpoB* gene was used as the endogenous reference control (primers listed in Table 2) and the relative transcript change was determined using the 2^-ΔΔCt^ relative quantification method [32]. Student’s *t*-test was used to calculate statistical significance (*P* < 0.05).

### Microarray procedures, hybridization and analysis

DNA microarray was performed using an Agilent custom-designed oligonucleotide arrays as previously described [33]. Briefly, cDNA probes were prepared from 5 μg of RNA pools obtained from MC58 or MC58 *ΔptsH* grown in GCB broth until mid-log and hybridized as described [33]. Three hybridizations per experiment were performed using cDNA probes from three independent pools. Differentially expressed genes were assessed by grouping all log_2_ ratios of the Cy5 and Cy3 values corresponding to each gene, within experimental replicas and spot replicas, and comparing them against the zero value by Student’s *t* test statistics (one tail). Microarray data are available in the ArrayExpress database (www.ebi.ac.uk/arrayexpress) under accession number E-MTAB-4997.

### H_2_O_2_ and anaerobic sensitivity assays

To evaluate sensitivity to oxidative stress, a disc assay with H_2_O_2_ was used. From overnight growth in GCB plates, a bacterial suspension of MC58, Δ*ptsH* and Δ*ptsH*-C_RBS_ strains was prepared in GCB broth with a final OD_600_ at 0.5. 1 ml of suspension was poured on the top of a square GCB agar plate. After 15 min of incubation at 37^a^C with 5% CO_2_, sterile disks containing 10 μl of either 0.3 or 3% of H_2_O_2_ solution were placed onto the plates. After overnight incubation, the diameter of the inhibition halo was measured. We also tested growth sensitivity of these *N*. *meningitidis* strains to anaerobic stress and ability to make nitrite respiration. Serial dilutions of bacteria grown in GCB medium, which had reached an OD_600_ of 0.5, were spotted on GCB plates with 10 mM NaHCO_3_ and 5 mM NaNO_2_. These plates were incubated overnight at 37°C in a 2.5 l anaerobic jar in the presence of an ANAEROGEN sachet (Thermo Scientific), generator of anaerobic environment. The following day, pictures of the plate were taken, and a scale of growth from 0–4 was defined with 0 corresponding to no growth and 4 a homogeneous spot of meningococcus growth. All experiments were done at least in duplicate. A two-way ANOVA multiple comparison test was carried out in order to assess statistically significant differences (GraphPad Prism version 6.0a for Mac, GraphPad Software, CA, USA).

### Statistical analysis

For mice experiments with non normal distribution we applied as nonparametric statistical test the two-tailed Mann-Whitney test. For multiple comparison experiments we used ANOVA 2 tails statistical analysis.

## Results

### HPr contributes to successful bacteremia in the mice infection model

**Fig 1:**
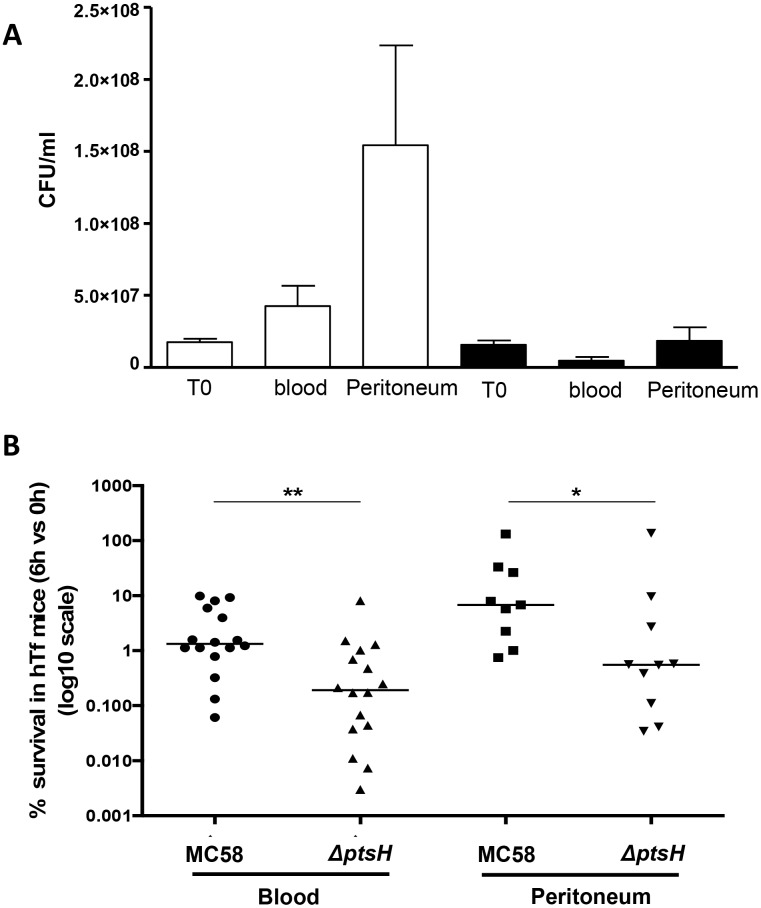
Survival of meningococci in transgenic mice expressing the human transferrin. Transgenic mice expressing hTf were injected intraperitoneally with 1x10^7^ bacteria. Bacterial colony forming units (CFU) per ml were determined from blood samples and peritoneum washes after 6 h post-infection. (A) The results are expressed in CFU/ml for the inoculum (T0) and for the bacteria loads in the blood and the peritoneal cavity after 6h of infection. (B) The data are expressed as percentage of bacteria survival in mice (CFU per ml recovered from samples at 6 h post-infection / CFU per ml in the initial inoculum) X100. The median ratio from at least three independent experiments is presented in the graph (statistical significances using two-tailed Mann-Whitney test, * *p* < 0.05, and ** *P* < 0.01).

We first constructed an HPr deletion mutant derived from strain MC58 that is a hyperinvasive isolate of the clonal complex cc32 [24]. These two strains were used to infect transgenic mice expressing hTf [22]. Mice were infected intraperitoneally with 10^7^ CFUs and 6 h post-infection bacterial counts were determined by serial dilutions and plating of both blood and peritoneal washes (see Material and Methods for details). As shown in Fig 1, strain MC58 provoked significantly higher levels of bacteremia in transgenic mice compared to its *ΔptsH* mutant. The bacterial load in peritoneum washes was also higher in mice infected with the wild-type strain compared to mice infected with the Δp*tsH* mutant (Fig 1). These results confirm the impact of HPr on experimental bacteremia [19]. The mutant did not show a growth defect relative to the wild type strain (S1 Fig). Its lower numbers in the blood therefore suggested that the Δp*tsH* mutant might be unable to cross the peritoneum to the blood and/or might undergo rapid clearance in the blood.

### Rapid clearance of the *ΔptsH* mutant in the bacteremia model

**Fig 2:**
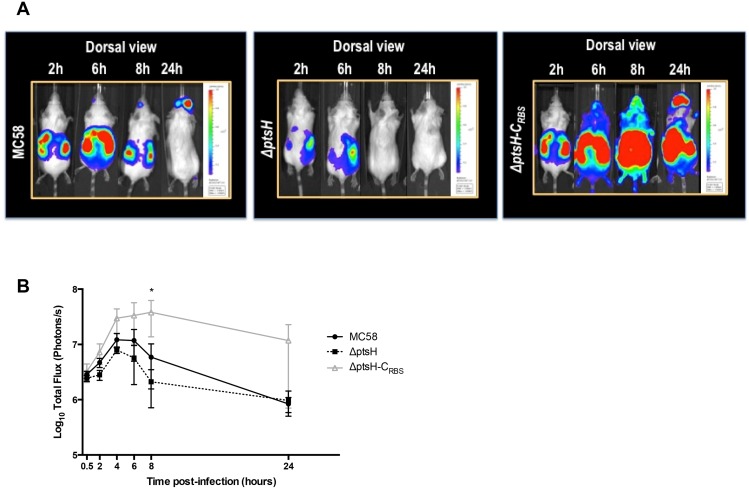
Dissemination profile of *N*. *meningitidis* infection in the transgenic human transferrin mouse model. Mice (at least 5 per group) were infected intraperitoneally with standardized inocula of bacteria and analyzed for bioluminescence at the indicated times. (A) Dorsal view of mice infected with MC58, Δ*ptsH*, and Δ*ptsH*-C_RBS_ strains at 2, 6, 8 and 24 h post-challenge. Images are of one mouse, representative of the group, and depict photographs overlaid with color representations of luminescence intensity, measured in total photons/sec and indicated on the scales, where red represents most intense and blue least intense luminescence. (B) Total body luminescence quantification. The total luminescence was quantified for each individual mouse using a region of interest corresponding to the whole mouse. Data from all mice for each strain at each time point were expressed as mean values ± SEM. The mean values and SEM of each strain were calculated from the results obtained with at least 5 infected mice.

In order to follow the meningococcal infection in the transgenic mice we engineered bioluminescent derivatives of the MC58 strain and its Δ*ptsH* mutant. We also constructed a complemented strain, which we called Δ*ptsH-*C_RBS_. We injected mice with similar bacterial load and using dynamic live imaging we followed *in vivo* the course of infection by the wild-type, the Δ*ptsH* mutant and the Δp*tsH-*C_RBS_ strain until 24 h post infection. As shown in Fig 2, all three strains caused a systemic infection in the transgenic mouse model expressing hTf. For the wild-type and the Δ*ptsH* mutant, maximal bioluminescence and hence systemic infection, occurred at 4–6 hours post infection (Fig 2B). However, the Δ*ptsH* mutant strain resulted generally in less luminescence and was also cleared faster than the wild-type. Complementation of the mutation restored full infectivity and the complemented mutant exhibited a prolonged and higher infection throughout the 24 h observed period.

In order to further understand the behavior of the Δ*ptsH* mutant we explored the local and systemic host innate immune responses. We characterized the recruitment of inflammatory cells at the initial site of infection (peritoneal washes) by flow cytometry (FACS) analysis at 6 h post-infection (S2 Fig). We also measured the production of pro-inflammatory cytokines TNF-alpha, IL-6 and KC at 2 h and 6 h post-infection both in the peritoneal washes and in blood samples (S3 Fig). As shown in both S2 and S3 Figs, there was no significant difference in the host immune response to the strains tested either locally, at the site of injection (peritoneum), or in the blood except for TNF-alpha that showed significant higher levels after 2 h of infection with the MC58 wild type strain. In conclusion, these data suggest that the Δp*tsH* mutant and the wild-type strain are able to cross the peritoneum and invade the blood system of the host.

### The *ΔptsH* strain is more susceptible to complement killing

**Fig 3:**
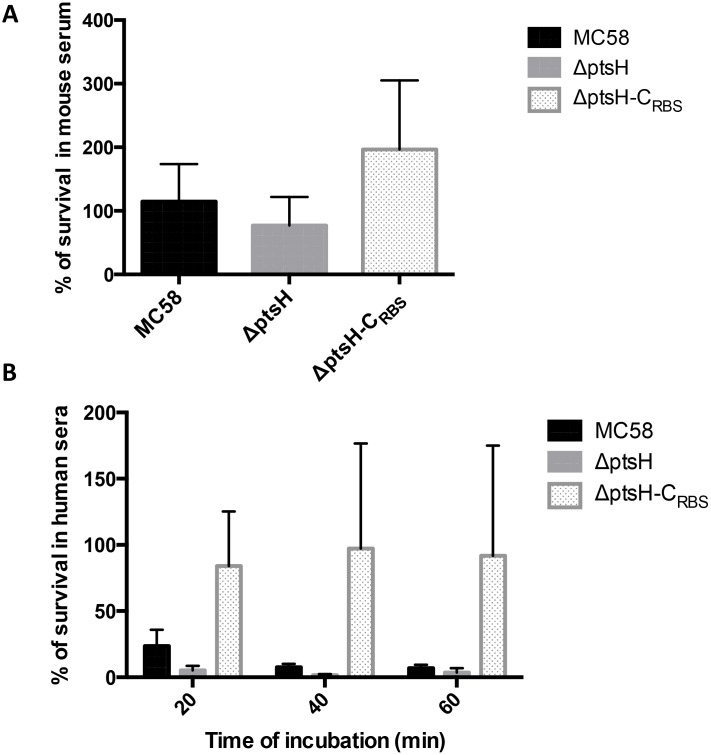
Meningococcal survival in serum. (A) Bacteria were incubated with MgCl_2_ + CaCl_2_-treated mouse serum (25% (v/v)) for 60 min at 37°C. (B) Bacteria were incubated with MgCl_2_ + CaCl_2_-treated human pooled serum (25% (v/v)) for 20, 40 and 60 min at 37°C. CFUs were determined as described in materials and methods and data represented are relative to the CFUs determined for the inoculum of each strain. Presented are the mean values and standard deviations of at least three independent experiments.

**Fig 4:**
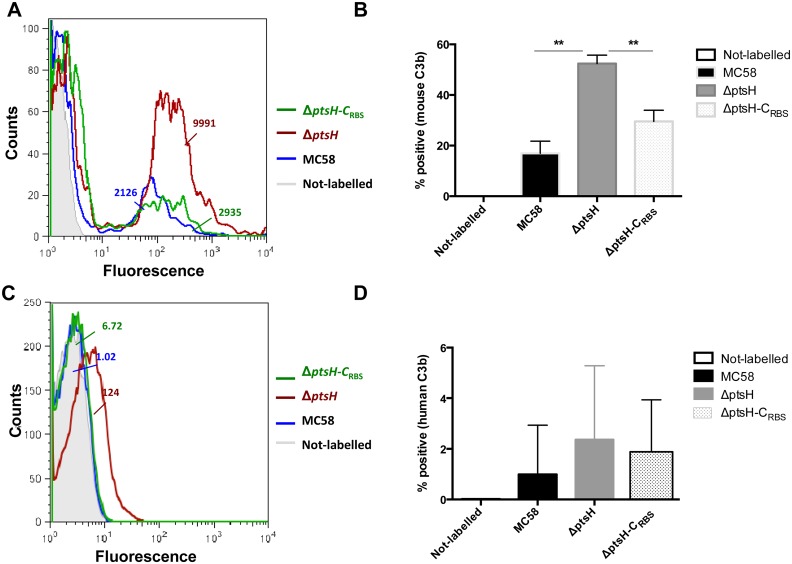
Flow cytometry analysis of complement activation by measuring C3b deposition. The *N*. *meningitidis* MC58, Δ*ptsH*, and Δ*ptsH*-C_RBS_ strains were incubated for 60 min or 30 min with 25% (v/v) of mouse serum or human pooled sera containing MgCl_2_+ CaCl_2_. (A and C). Histograms from representative experiments of mouse and human C3b deposition are shown in panels A and C respectively. The index of mean median fluorescence intensity is shown (MFI index) (the text color corresponds to the color of the histogram). (B) The percentage of positive events for mouse C3 fragment deposition from pooled experiments is shown. Each bar represents the mean (standard error of the mean) of at least two independent experiments. ** *P* < 0.01 (two-tailed Mann-Whitney test). (D) The percentage of positive events for human C3 fragment deposition from pooled experiments is shown. Each bar represents the mean (standard error of the mean) of at least three independent experiments.

We next tested meningococcal survival in mouse sera and pooled normal human sera (NHS). No killing was observed in heat-inactivated serum. While all three strains were resistant to incubation in 25% mouse sera, with only minor reduction (80% survival) for the Δp*tsH* mutant (Fig 3A), in the presence of human sera the mutant exhibited significantly increased sensitivity. As seen in Fig 3B, after 20 min of incubation only about 10% of the Δ*ptsH* cells survived compared to 25% of the wild-type and 80% of the complemented strain. Both Δ*ptsH* mutant and MC58 wild type strains showed similar survival after 40 and 60 min of incubation while the complemented strain showed higher survival rates at all time points. In the complemented strain, *ptsH* expression is under the control of the constitutive P_tac_ promoter (see Material and Methods) being probably overespressed (S4 Fig). These results suggest that in the absence of a functional HPr protein the mutant is more susceptible to complement-mediated killing and that overproduction of HPr even protects *N*. *meningitidis*. We therefore tested the complement activation at the bacterial surface by measuring the C3b deposition by FACS analysis. In Fig 4A and 4B the Δ*ptsH* mutant strain showed higher deposition of mouse C3b than the wild-type or the complemented strains. Similar results were also obtained with NHS (Fig 4C), further suggesting that the complement is the factor responsible for the rapid clearance of the Δ*ptsH* mutant in the blood.

### Deletion of *ptsH* does not affect the expression of known meningococcal factors involved in resistance to complement-mediated killing

**Fig 5:**
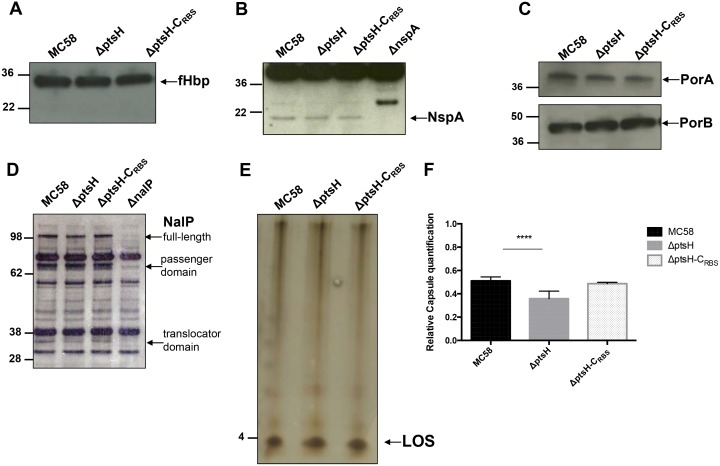
Known meningococcal complement resistance factors are not affected by the lack of HPr, with the exception of capsule. Total lysates of wild-type (MC58), Δ*ptsH*, and Δ*ptsH*-C_RBS_ strains were prepared and proteins were separated on a 4–12% Bis-Tris gel, transferred to a nitrocellulose membrane and detected with various antibodies. Determination of production levels of (A) factor H binding protein (fHbp) and (B) of Neisserial protein A (NspA). Strain Δ*nspA* was used as a negative control. (C) Production levels of Porin A (PorA) and Porin B (PorB). (D) Production levels of NalP. The Δ*nalP* mutant was used as a negative control. NalP can undertake autoproteolytic processing resulting in two forms: the passenger domain and the translocator domain are indicated. (E) Silver stained Tris-Glycine SDS-PAGE LPS gel of proteinase K-treated whole cells of *N*. *meningitidis* isolates. (F) Relative capsule quantification of MC58, Δ*ptsH*, and Δ*ptsH*-C_RBS_ strains from ELISA titers.

The complement-mediated killing system is one of the major mechanisms that the host uses in order to eliminate *N*. *meningitidis* [5]. Nevertheless, meningococci possess several surface structures that allow it to subvert complement-mediated killing [34]. We therefore analyzed the levels of these structures in the MC58 wild-type strain, its Δp*tsH* mutant and the Δp*tsH-*C_RBS_ complemented strain [5, 13]. Western blot analysis revealed no detectable differences among these strains for the following proteins fHbp, NspA, PorA, PorB, NalP (Fig 5A–5D, respectively). Extracted lipooligosaccharide (LOS) did not show any change in electrophoretic mobility (Fig 5). Moreover, when LOS from MC58, the Δp*tsH* mutant and the Δp*tsH-*C_RBS_ complemented strains was analyzed in PAGE against polyclonal anti-LOS B, 7, 9 no differences could be observed. Thus, we could also exclude lipid A modification since they would lead to changes in electrophoretic mobility or to the abolishment of LOS detection (data not shown)[35, 36]. The production of capsule measured by ELISA titers was slightly but significantly lower in the Δ*ptsH* mutant compared to the wild-type or complemented strains. These data were also confirmed by dot blotting analysis (S5 Fig). All these results, including the slight capsule reduction, suggested a not yet described mechanism that may be involved in the reduced virulence of the Δ*ptsH* mutant.

### Transcriptional profiling with wild-type and Δ*ptsH* reveals changes in stress response

In order to assess global differences in gene expression that may be regulated directly or indirectly by HPr, a global transcriptomic profile analysis of the Δ*ptsH* mutant and the wild-type MC58 strain was performed. RNA of wild-type and Δ*ptsH* mutant grown in rich GCB broth until mid-logarithmic phase was extracted and differential gene expression through microarray analysis was performed. RNA was extracted from mid-log phase, which reflects the exponential growth observed during septicemia infection in the animal model and also avoids autolysis effects occurring during late growth phase. We observed that 49 genes were differently regulated in the absence of HPr. Of these, 41 genes were up-regulated and only 8 genes were down-regulated. Besides *ptsH*, the other down-regulated genes corresponded to sulfate transport system (NMB0880, NMB0881), two hypothetical proteins (NMB0120, NMB0508), a conserved hypothetical integral membrane protein (NMB2140), and the modulators of drug activity B (NMB1857, NMB0977). The up-regulated genes in the absence of *ptsH* corresponded mainly to hypothetical proteins (44%) and proteins involved in posttranslational modification, protein turnover, and stress resistance, such as *dnaJ*, *dnaK*, *grpE*, *clpB*, *groL* and *hslO*. In addition, the genes of two transcriptional regulators, *rpoD* (NMB1538) and *gdhR* (NMB1563) involved in exponential phase transcription and carbon metabolism respectively, were found up-regulated as well as several proteins involved in redox reactions (Table 3). In order to confirm the results obtained in the microarray expression profiling, we selected a subset of 8 genes being either up- or down-regulated and performed real-time quantitative PCR (qRT-PCR) (S6A Fig). The results obtained are similar to the microarray data with a good coefficient of correlation (r2 = 0.909) (S6B Fig). These data suggest that HPr has pleiotropic effects on the expression of genes (HPr regulon) that are involved in various biological processes in meningococci.

**Table 3:** List of genes regulated in Δ*ptsH* mutant in MC58 background.

*ptsH* KO/MC58 in GCB medium					
Gene Symbol	Gene	Product	Class Description*	log_2_ ratio^a^	p-value^b^
NMB2045	*ptsH*	phosphocarrier protein HPr (phosphotransferase system, histidine-containing protein)	Carbohydrate transport and metabolism	-3.22	0.04
NMB0120		hypothetical protein	Function unknown	-2.55	0.01
NMB0880	*cysW*	sulfate transport system permease protein CysW	Inorganic ion transport and metabolism	-1.46	0.02
NMB0881	*cysT*	sulfate transport system permease protein CysT	Inorganic ion transport and metabolism	-1.22	0.02
NMB1857		putative NADPH-quinone dehydrogenase (modulator of drug activity B)	General function prediction only	-1.21	0.00
NMB0508		hypothetical protein	Function unknown	-1.20	0.00
NMB0977		putative NADPH oxidoreductase (modulator of drug activity B)	General function prediction only	-1.20	0.05
NMB2140		conserved hypothetical integral membrane protein	Function unknown	-1.06	0.00
NMB0791	*ppiB*	peptidyl-prolyl cis-trans isomerase B (PPIase B; rotamase B)	Posttranslational modification, protein turnover, chaperones	1.00	0.00
NMB0213		putative Sm-like integral membrane protein	Cell wall/membrane/envelope biogenesis	1.00	0.00
NMB1972	*groL*	60 kDa chaperonin (protein Cpn60; GroEL protein; 63 kDa stress protein; GSP63; HSP60)	Posttranslational modification, protein turnover, chaperones	1.03	0.00
NMB0216	*katA*	catalase	Inorganic ion transport and metabolism	1.03	0.02
NMB0946		putative peroxiredoxin (thioredoxin reductase)	Posttranslational modification, protein turnover, chaperones	1.08	0.00
NMB1369		hypothetical protein	Function unknown	1.09	0.00
NMB1055	*glyA*	serine hydroxymethyltransferase (serine methylase; SHMT)	Amino acid transport and metabolism	1.10	0.01
NMB0716		truncated cell volume regulation protein A homolog (C-terminal 16% of the protein)	Inorganic ion transport and metabolism	1.12	0.02
NMB0558		hypothetical protein	Function unknown	1.13	0.00
NMB1375		putative type III restriction-modification system enzyme Mod (pseudogene part 1)	Replication, recombination and repair	1.27	0.03
NMB1272		hypothetical protein	Function unknown	1.27	0.00
NMB1562		conserved hypothetical integral membrane protein	Function unknown	1.28	0.00
NMB1370		hypothetical protein	Function unknown	1.38	0.00
NMB0559		putative ubiquinone biosynthesis protein UbiB	Signal transduction mechanisms	1.39	0.00
NMB1538	*rpoD*	RNA polymerase sigma factor RpoD (Sigma-70)	Transcription	1.44	0.00
NMB1564		putative OsmC-like protein	Posttranslational modification, protein turnover, chaperones	1.44	0.00
NMB0715		truncated cell volume regulation protein A homolog (N-terminal 7% of the protein)	Inorganic ion transport and metabolism	1.47	0.00
NMB1644		putative GTP-binding protein	General function prediction only	1.49	0.00
NMB0554	*dnaK*	chaperone protein DnaK (heat shock protein 70; heat shock 70 kDa protein; HSP70)	Posttranslational modification, protein turnover, chaperones	1.57	0.00
NMB0947	*lpdA2*	dihydrolipoyl dehydrogenase (E3 component of pyruvate complex; dihydrolipoamide dehydrogenase)	Energy production and conversion	1.63	0.00
NMB1563	*gdhR*	putative HTH-type transcriptional regulator	Transcription	1.65	0.00
NMB1475		conserved hypothetical periplasmic protein	Energy production and conversion	1.67	0.01
NMB0557		conserved hypothetical protein	Function unknown	1.70	0.00
NMB1231	*lon*	ATP-dependent protease Lon	Posttranslational modification, protein turnover, chaperones	1.78	0.00
NMB1376		putative type III restriction-modification system enzyme Res (pseudogene part 1)	Defense mechanisms	1.87	0.03
NMB1335		conserved hypothetical protein	Function unknown	1.94	0.00
NMB1334		hypothetical protein	Function unknown	1.94	0.00
NMB0059	*dnaJ*	chaperone protein DnaJ	Posttranslational modification, protein turnover, chaperones	1.99	0.00
NMB0907		hypothetical protein	Function unknown	2.00	0.00
NMB1336		conserved hypothetical protein	Function unknown	2.06	0.00
NMB0906		conserved hypothetical protein	Function unknown	2.08	0.00
NMB0552		conserved hypothetical integral membrane protein	Inorganic ion transport and metabolism	2.17	0.00
NMB0901		D-lactate dehydrogenase-like protein	Energy production and conversion	2.20	0.00
NMB2000	*hslO*	33 kDa chaperonin (heat shock protein 33 homolog; HSP33)	Posttranslational modification, protein turnover, chaperones	2.27	0.00
NMB0904		hypothetical protein	Function unknown	2.27	0.00
NMB0905		hypothetical protein	Function unknown	2.27	0.00
NMB1337		conserved hypothetical protein	Function unknown	2.28	0.00
NMB0902		hypothetical protein	Function unknown	2.33	0.00
NMB0903		hypothetical protein	Function unknown	2.45	0.00
NMB0561	*grpE*	protein GrpE (HSP-70 cofactor)	Posttranslational modification, protein turnover, chaperones	2.89	0.00
NMB1472	*clpB*	chaperone ClpB (short)	Posttranslational modification, protein turnover, chaperones	3.23	0.00

^a^ Genes- up- and down-regulated were selected setting log2 ratio cut-off ≥ 1 and ≤ -1, respectively.

^b^ P-value cut-off was set ≤ 0.05.

* according to Clusters of Orthologous Groups (COGS) database classification

#### HPr is important for stress resistance

**Fig 6:**
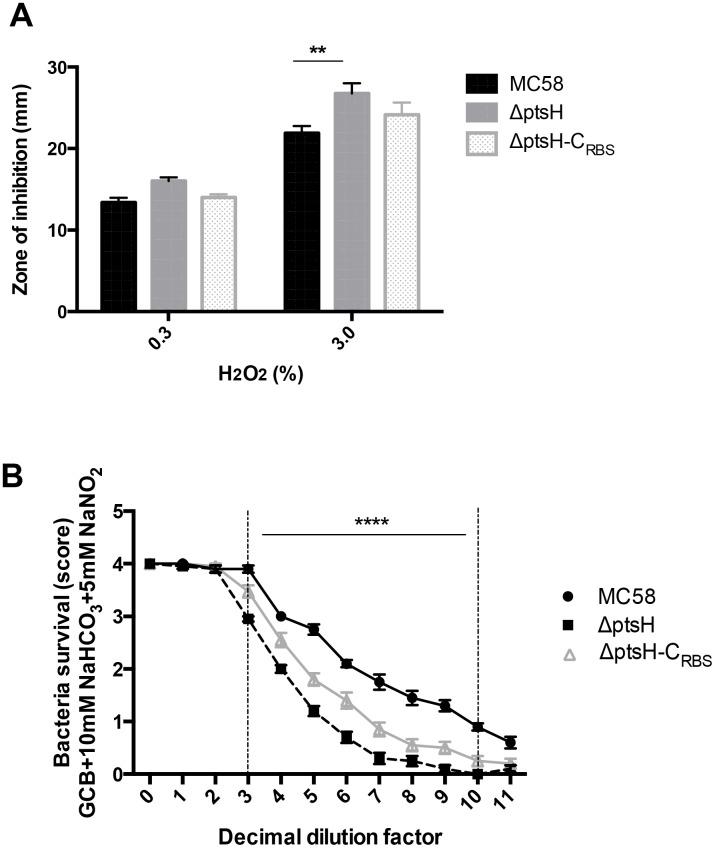
*N*. *meningitidis* lacking HPr is more sensitive to stress conditions. (**A**) Disc assays of bacterial sensitivity after exposure to 0.3 and 3% H_2_O_2_ were carried out with MC58 (wild-type), Δ*ptsH*, and Δ*ptsH*-C_RBS_ strains. Each bar represents the mean (with standard error of mean) of two independent experiments done in quadruplicate. (** *P* < 0.01 compared to the wild-type strain). (**B**) Serial dilution plate assays were performed to compare the tolerance to oxygen depletion and use of the denitrification pathway of MC58 (wild-type) to the Δ*ptsH* and Δ*ptsH*-C_RBS_ strains in the presence of nitrite. Presented are the results from three independent experiments carried out in duplicate (**** *P* < 0.0001 Δ*ptsH* compared to the wild-type strain).

Since several genes implicated in stress response were found to be up-regulated in mid-log growth phase in the absence of HPr, we tested the ability of the Δ*ptsH* mutant to survive under stress conditions, such as oxidative stress or oxygen depletion environments, that *N*. *meningitidis* could encounter during host infection [18]. As shown in Fig 6A the diameter of growth inhibition zone in the presence of H_2_O_2_ was higher for the Δ*ptsH* mutant compared to both wild-type and to complemented strains, especially at 3% of H_2_O_2_ where a statistically significant difference could be observed. This result suggests a higher susceptibility of the Δ*ptsH* mutant to oxidative stress. Furthermore, we observed that both the wild-type and complemented strains survived better under oxygen limitation in the presence of nitrite than the Δ*ptsH* mutant (Fig 6B and S7 Fig). These results suggest that, under oxidative stress or oxygen limitation, HPr is required for meningococcal response to these stress conditions.

## Discussion

The links between metabolism and bacterial virulence have been increasingly studied [18, 21]. They revealed a growing family of moonlighting multitask proteins that are frequently metabolic enzymes with a role in bacterial virulence [37]. It is the case of the elongation factor EF-Tu in *Leptospira*, which was shown to bind plasminogen and fH mediating C3b complement component degradation on the surface of leptospires and to contribute to complement inactivation [38]. In *N*. *meningitidis*, the fructose-1,6-bisphosphate aldolase was shown to be surface located and to contribute to cell adhesion [39]. HPr, a phosphocarrier protein of the PTS, has previously been suggested to be involved in meningococcal virulence on the basis of results in animal models [19]. Here we show that in the hyper-invasive MC58 strain, the HPr plays an important role in meningococcal survival during infection especially in blood, which is most likely through enhancing the degradation of C3b to subvert complement activation. Moreover, the reduced virulence of the Δ*ptsH* mutant does not seem to be due to lower invasiveness, as it was able to cross the peritoneum to the blood and to provoke the production of inflammatory cytokine levels similar to the wild-type and complemented strains. The lower virulence of Δ*ptsH* strain is most likely due to a rapid clearance in the blood.

In our model of transgenic mice expressing the human transferrin, all tested strains were able to infect mice by intra-peritoneal route with production of inflammatory cytokines. The higher levels of TNF-alpha that was observed 2 h after the infection with the wild type MC58 strain may be due to different physiological state of bacteria on the cultured plates. This difference disappeared after 6 h when bacteria had been adapted to their new environment. However, lower survival of meningococci in blood was observed when the *ptsH* gene was lacking. Complementation with a wild-type *ptsH* gene restored virulence although to a higher level. This virulence enhancement upon complementation was also observed in mouse and human sera survival assays. It is most likely due to a higher level of expression of the *ptsH* gene, which is under control of a stronger heterologous promoter (P_tac_) (S4 Fig) [9]. *N*. *meningitidis* has developed strategies to evade killing by complement by hijacking host complement regulatory proteins and use these molecules to downregulate complete activation [5]. However, these mechanisms developed by *N*. *meningitidis* are specific to human complement components. The complement activation on meningococci is inhibited by the human Factor H [5]. It is therefore anticipated that the overall deposition of C3b is higher in the mouse model than in the human sera [40–42]. Apart from a slight reduction in the capsule production, the mechanism of this activity remains to be determined, as it does not seem to use the known meningococcal factors involved in resistance to complement lysis such as fHbp or NspA [43, 44].

Our data also showed that the HPr protein is involved in resistance to stress conditions encountered in blood, like the production of reactive oxygen species (ROS) such as H_2_O_2_ through oxidative burst [45]. Moreover, not only does blood constitute an immunologically challenging compartment but it is also an oxygen-limiting environment as oxygen is linked to hemoglobin, and the denitrification pathway enables meningococcal survival via anaerobic respiration [46, 47]. The denitrification process has an important physiological role in meningococcus, as it allows NO detoxification and supports energy conservation through respiration [48]. We observed *in vitro*, that strains lacking HPr are more susceptible to H_2_O_2_ and defective in their ability to grow under anaerobic conditions compared to the wild-type and complemented strains. Our data suggests therefore that HPr plays a central and pleiotropic role in meningococcal survival in blood. Indeed, the transcriptomic analysis confirmed this conclusion and identified 49 differentially expressed genes, therefore resulting in a complex *N*. *meningitidis* pattern response to environment conditions. Most of the genes belonging to the HPr regulon correspond to stress response genes and hypothetical proteins, (some membrane-associated), suggesting a potential role during host-interaction.

These proteins may contribute to a complex network of host-bacteria-interaction to allow meningococcal survival. Moreover, since the transcriptomic analysis was carried out using microarrays, we cannot exclude that the HPr regulon may be even more complex including regulation of sRNA species. To prove this hypothesis, the next step would be to carry out a RNA-seq analysis. At the opposite, more stringent cut-off in the microarray analysis (for example log2 ratio cut-off ≥ 1.5 fold) may reduce the number of gene in the HPr regulon. Physiological analysis of these genes may be required to explore this regulon and its significance. Due to the diversity of meningococcal isolates, it would be interesting to extend our observation to other invasive and carriage isolates. In the meantime, the involvement of HPr in virulence has been suggested in two other different isolates in addition to strain MC58 (C311 a serogroup B strain and 2C4.3 a serogroup C strain) [17, 19]. The PTS component HPr is a multitasking protein involved in carbohydrate uptake and regulation of virulence factors in response to carbon source availability [20]. In *N*. *meningitidis* it seems to play functions in addition to those known for other Gram-negative bacteria and firmicutes [20]. Recently, we have reported that HPr was found to interact with the transcription regulator CrgA and its deletion affects virulence and capsule synthesis of *N*. *meningitidis* strain 2C4.3 [17].

We therefore suggest that HPr to be a member of the moonlighting proteins that may have evolved under selection (evolution probably by tinkering) [49]. Moreover, we have shown that HPr plays an important role against complement-mediated bacteriolysis probably by remodeling the action of several proteins.

Metabolism and virulence are not separated activities within bacteria, but often interlinked through biosynthetic pathways in which acquisition of key nutrients available in vivo serves to increase the fitness of the pathogen by enhancing its virulence through the avoidance of clearance by host immunity.

## Supporting Information

S1 Fig (PDF)

S2 Fig (PDF)

S3 Fig (PDF)

S4 Fig (PDF)

S5 Fig (PDF)

S6 Fig (PDF)

S7 Fig (PDF)

## References

[1] Yazdankhah SP, Caugant DA. *Neisseria meningitidis*: an overview of the carriage state. J Med Microbiol. 2004;53(Pt 9):821–32. . 15314188 10.1099/jmm.0.45529-0

[2] Caugant DA, Tzanakaki G, Kriz P. Lessons from meningococcal carriage studies. FEMS Microbiol Rev. 2007;31(1):52–63. . 17233635 10.1111/j.1574-6976.2006.00052.x

[3] Caugant DA, Maiden MC. Meningococcal carriage and disease—population biology and evolution. Vaccine. 2009;27 Suppl 2:B64–70. Epub 2009/05/26. 10.1016/j.vaccine.2009.04.061 S0264-410X(09)00615-X [pii]. 19464092 PMC2719693

[4] Rosenstein NE, Perkins BA, Stephens DS, Popovic T, Hughes JM. Meningococcal disease. N Engl J Med. 2001;344(18):1378–88. . 11333996 10.1056/NEJM200105033441807

[5] Lewis LA, Ram S. Meningococcal disease and the complement system. Virulence. 2014;5(1):98–126. Epub 2013/10/10. 10.4161/viru.26515 26515 [pii]. 24104403 PMC3916388

[6] Geoffroy MC, Floquet S, Metais A, Nassif X, Pelicic V. Large-scale analysis of the meningococcus genome by gene disruption: resistance to complement-mediated lysis. Genome Res. 2003;13(3):391–8. . 12618369 10.1101/gr.664303 PMC430250

[7] Zarantonelli ML, Szatanik M, Giorgini D, Hong E, Huerre M, Guillou F, et al Transgenic mice expressing human transferrin as a model for meningococcal infection. Infect Immun. 2007;75(12):5609–14. . 17893132 10.1128/IAI.00781-07 PMC2168318

[8] Madico G, Ngampasutadol J, Gulati S, Vogel U, Rice PA, Ram S. Factor H binding and function in sialylated pathogenic *Neisseriae* is influenced by gonococcal, but not meningococcal, porin. J Immunol. 2007;178(7):4489–97. Epub 2007/03/21. 178/7/4489 [pii]. . 17372007 10.4049/jimmunol.178.7.4489

[9] Seib KL, Serruto D, Oriente F, Delany I, Adu-Bobie J, Veggi D, et al Factor H-binding protein is important for meningococcal survival in human whole blood and serum and in the presence of the antimicrobial peptide LL-37. Infect Immun. 2009;77(1):292–9. Epub 2008/10/15. 10.1128/IAI.01071-08 IAI.01071-08 [pii]. 18852235 PMC2612274

[10] Lewis LA, Carter M, Ram S. The relative roles of factor H binding protein, neisserial surface protein A, and lipooligosaccharide sialylation in regulation of the alternative pathway of complement on meningococci. J Immunol. 2012;188(10):5063–72. Epub 2012/04/17. 10.4049/jimmunol.1103748 jimmunol.1103748 [pii]. 22504643 PMC3345070

[11] Lewis LA, Ngampasutadol J, Wallace R, Reid JE, Vogel U, Ram S. The meningococcal vaccine candidate neisserial surface protein A (NspA) binds to factor H and enhances meningococcal resistance to complement. PLoS Pathog. 2010;6(7):e1001027 Epub 2010/08/06. 10.1371/journal.ppat.1001027 20686663 PMC2912398

[12] Lewis LA, Vu DM, Granoff DM, Ram S. Inhibition of the alternative pathway of nonhuman infant complement by porin B2 contributes to virulence of *Neisseria meningitidis* in the infant rat model. Infect Immun. 2014;82(6):2574–84. Epub 2014/04/02. 10.1128/IAI.01517-14 IAI.01517-14 [pii]. 24686052 PMC4019150

[13] Del Tordello E, Vacca I, Ram S, Rappuoli R, Serruto D. *Neisseria meningitidis* NalP cleaves human complement C3, facilitating degradation of C3b and survival in human serum. Proc Natl Acad Sci U S A. 2014;111(1):427–32. Epub 2013/12/25. 10.1073/pnas.1321556111[pii]. 24367091 PMC3890809

[14] Mallavia LP, Weiss E. Catabolic activities of *Neisseria meningitidis*: utilization of glutamate. J Bacteriol. 1970;101(1):127–32. Epub 1970/01/01. 4983643 10.1128/jb.101.1.127-132.1970 PMC250459

[15] Leighton MP, Kelly DJ, Williamson MP, Shaw JG. An NMR and enzyme study of the carbon metabolism of *Neisseria meningitidis*. Microbiology. 2001;147(Pt 6):1473–82. Epub 2001/06/08. . 11390678 10.1099/00221287-147-6-1473

[16] Smith H, Tang CM, Exley RM. Effect of host lactate on gonococci and meningococci: new concepts on the role of metabolites in pathogenicity. Infect Immun. 2007;75(9):4190–8. Epub 2007/06/15. IAI.00117-07 [pii] 10.1128/IAI.00117-07 17562766 PMC1951187

[17] Derkaoui M, Antunes A, Poncet S, Abdallah JN, Joyet P, Maze A, et al The phosphocarrier protein HPr of *Neisseria meningitidis* interacts with the transcription regulator CrgA and its deletion affects capsule production, cell adhesion and virulence. Mol Microbiol. 2016 Epub 2016/02/10. 10.1111/mmi.13349 . 26858137

[18] Schoen C, Kischkies L, Elias J, Ampattu BJ. Metabolism and virulence in *Neisseria meningitidis*. Front Cell Infect Microbiol. 2014;4:114 Epub 2014/09/06. 10.3389/fcimb.2014.00114 25191646 PMC4138514

[19] Sun YH, Bakshi S, Chalmers R, Tang CM. Functional genomics of *Neisseria meningitidis* pathogenesis. Nat Med. 2000;6(11):1269–73. . 11062540 10.1038/81380

[20] Deutscher J, Ake FM, Derkaoui M, Zebre AC, Cao TN, Bouraoui H, et al The bacterial phosphoenolpyruvate:carbohydrate phosphotransferase system: regulation by protein phosphorylation and phosphorylation-dependent protein-protein interactions. Microbiol Mol Biol Rev. 2014;78(2):231–56. Epub 2014/05/23. [pii]. 24847021 10.1128/MMBR.00001-14 PMC4054256

[21] Deutscher J, Francke C, Postma PW. How phosphotransferase system-related protein phosphorylation regulates carbohydrate metabolism in bacteria. Microbiol Mol Biol Rev. 2006;70(4):939–1031. Epub 2006/12/13. 70/4/939 [pii] 10.1128/MMBR.00024-06 17158705 PMC1698508

[22] Szatanik M, Hong E, Ruckly C, Ledroit M, Giorgini D, Jopek K, et al Experimental meningococcal sepsis in congenic transgenic mice expressing human transferrin. PLoS One. 2011;6(7):e22210 Epub 2011/08/04. 10.1371/journal.pone.0022210 PONE-D-11-04411 [pii]. 21811575 PMC3141004

[23] Kellogg DS Jr., Peacock WL Jr., Deacon WE, Brown L, Pirkle DI. *Neisseria gonorrhoeae*. I. Virulence Genetically Linked to Clonal Variation. J Bacteriol. 1963;85:1274–9. . 14047217 10.1128/jb.85.6.1274-1279.1963 PMC278328

[24] Tettelin H, Saunders NJ, Heidelberg J, Jeffries AC, Nelson KE, Eisen JA, et al Complete genome sequence of *Neisseria meningitidis* serogroup B strain MC58. Science. 2000;287(5459):1809–15. . 10710307 10.1126/science.287.5459.1809

[25] Guiddir T, Deghmane AE, Giorgini D, Taha MK. Lipocalin 2 in cerebrospinal fluid as a marker of acute bacterial meningitis. BMC Infect Dis. 2014;14(1):276 Epub 2014/06/03. 10.1186/1471-2334-14-276 1471-2334-14-276 [pii]. 24885531 PMC4033677

[26] Hanahan D. Studies on transformation of Escherichia coli with plasmids. J Mol Biol. 1983;166(4):557–80. . 6345791 10.1016/s0022-2836(83)80284-8

[27] Miller JH. Experiments in Molecular Genetics. Cold Spring Harbor, NY: Cold Spring Harbor Laboratory; 1972.

[28] Sambrook J, Fritsch EF, Maniatis T. Molecular cloning: A laboratory Manual. Cold Spring Harbor, N.Y: Cold Spring Harbor Laboratory Press; 1989.

[29] Hong E, Giorgini D, Deghmane AE, Taha MK. Functional impacts of the diversity of the meningococcal factor H binding protein. Vaccine. 2012;31:183–9. Epub 2012/11/06. S0264-410X(12)01532-0 [pii] 10.1016/j.vaccine.2012.10.072 . 23123023

[30] Abdillahi H, Poolman JT. *Neisseria meningitidis* group B serosubtyping using monoclonal antibodies in whole-cell ELISA. Microb Pathog. 1988;4(1):27–32. . 3143890 10.1016/0882-4010(88)90045-9

[31] Hitchcock PJ, Brown TM. Morphological heterogeneity among *Salmonella* lipopolysaccharide chemotypes in silver-stained polyacrylamide gels. J Bacteriol. 1983;154(1):269–77. . 6187729 10.1128/jb.154.1.269-277.1983 PMC217456

[32] Livak KJ, Schmittgen TD. Analysis of relative gene expression data using real-time quantitative PCR and the 2(-Delta Delta C(T)) Method. Methods. 2001;25(4):402–8. . 11846609 10.1006/meth.2001.1262

[33] Fantappie L, Metruccio MM, Seib KL, Oriente F, Cartocci E, Ferlicca F, et al The RNA chaperone Hfq is involved in stress response and virulence in *Neisseria meningitidis* and is a pleiotropic regulator of protein expression. Infect Immun. 2009;77(5):1842–53. Epub 2009/02/19. 10.1128/IAI.01216-08 IAI.01216-08 [pii]. 19223479 PMC2681778

[34] Pizza M, Rappuoli R. *Neisseria meningitidis*: pathogenesis and immunity. Curr Opin Microbiol. 2015;23:68–72. Epub 2014/12/03. 10.1016/j.mib.2014.11.006 S1369-5274(14)00165-9 [pii]. . 25461575

[35] Zarantonelli ML, Carlier JP, Alonso JM, Taha MK. Insertional inactivation of the *lpxA* gene involved in the biosynthesis of lipid A in *Neisseria meningitidis* resulted in *lpxA/lpxA*::*aph-3'* heterodiploids. FEMS Microbiol Lett. 2003;226(1):51–6. . 13129607 10.1016/S0378-1097(03)00558-5

[36] Steeghs L, den Hartog R, den Boer A, Zomer B, Roholl P, van der Ley P. Meningitis bacterium is viable without endotoxin. Nature. 1998;392(6675):449–50. . 9548250 10.1038/33046

[37] Henderson B, Martin A. Bacterial moonlighting proteins and bacterial virulence. Curr Top Microbiol Immunol. 2013;358:155–213. Epub 2011/12/07. 10.1007/82_2011_188 . 22143554

[38] Wolff DG, Castiblanco-Valencia MM, Abe CM, Monaris D, Morais ZM, Souza GO, et al Interaction of Leptospira elongation factor Tu with plasminogen and complement factor H: a metabolic leptospiral protein with moonlighting activities. PLoS One. 2013;8(11):e81818 Epub 2013/12/07. 10.1371/journal.pone.0081818 PONE-D-13-28961 [pii]. 24312361 PMC3842364

[39] Tunio SA, Oldfield NJ, Berry A, Ala'Aldeen DA, Wooldridge KG, Turner DP. The moonlighting protein fructose-1, 6-bisphosphate aldolase of *Neisseria meningitidis*: surface localization and role in host cell adhesion. Mol Microbiol. 2010;76(3):605–15. 10.1111/j.1365-2958.2010.07098.x 20199602

[40] Schneider MC, Exley RM, Ram S, Sim RB, Tang CM. Interactions between *Neisseria meningitidis* and the complement system. Trends Microbiol. 2007;15(5):233–40. . 17398100 10.1016/j.tim.2007.03.005

[41] Costa I, Pajon R, Granoff DM. Human factor H (FH) impairs protective meningococcal anti-FHbp antibody responses and the antibodies enhance FH binding. MBio. 2014;5(5):e01625–14. Epub 2014/08/28. 10.1128/mBio.01625-14 e01625-14 [pii] mBio.01625-14 [pii]. 25161192 PMC4173785

[42] Taha M-K, Claus H, Lappann M, Veyrier FJ, Otto A, Becher D, et al Evolutionary events associated with an outbreak of meningococcal disease in men who have sex with men. PLoS One. 2016. 10.1371/journal.pone.0154047 PMC4864352 27167067

[43] Vu DM, Shaughnessy J, Lewis LA, Ram S, Rice PA, Granoff DM. Enhanced bacteremia in human factor H transgenic rats infected by *Neisseria meningitidis*. Infect Immun. 2012;80(2):643–50. Epub 2011/11/23. IAI.05604-11 [pii] 10.1128/IAI.05604-11 22104107 PMC3264313

[44] Giuntini S, Vu DM, Granoff DM. fH-dependent complement evasion by disease-causing meningococcal strains with absent fHbp genes or frameshift mutations. Vaccine. 2013;31(38):4192–9. Epub 2013/06/25. 10.1016/j.vaccine.2013.06.009 S0264-410X(13)00778-0 [pii]. 23791680 PMC3756549

[45] Urban CF, Lourido S, Zychlinsky A. How do microbes evade neutrophil killing? Cell Microbiol. 2006;8(11):1687–96. Epub 2006/08/31. CMI792 [pii] 10.1111/j.1462-5822.2006.00792.x . 16939535

[46] Anjum MF, Stevanin TM, Read RC, Moir JW. Nitric oxide metabolism in *Neisseria meningitidis*. J Bacteriol. 2002;184(11):2987–93. Epub 2002/05/11. 12003939 10.1128/JB.184.11.2987-2993.2002 PMC135047

[47] Laver JR, Stevanin TM, Messenger SL, Lunn AD, Lee ME, Moir JW, et al Bacterial nitric oxide detoxification prevents host cell S-nitrosothiol formation: a novel mechanism of bacterial pathogenesis. FASEB J. 2010;24(1):286–95. Epub 2009/09/02. 10.1096/fj.08-128330 fj.08-128330 [pii]. 19720623 PMC2820398

[48] Rock JD, Thomson MJ, Read RC, Moir JW. Regulation of denitrification genes in *Neisseria meningitidis* by nitric oxide and the repressor NsrR. J Bacteriol. 2007;189(3):1138–44. Epub 2006/11/24. JB.01368-06 [pii] 10.1128/JB.01368-06 17122348 PMC1797324

[49] Jacob F. Evolution and tinkering. Science. 1977;196(4295):1161–6. . 860134 10.1126/science.860134

[50] Echenique-Rivera H, Muzzi A, Del Tordello E, Seib KL, Francois P, Rappuoli R, et al Transcriptome analysis of *Neisseria meningitidis* in human whole blood and mutagenesis studies identify virulence factors involved in blood survival. PLoS Pathog. 2011;7(5):e1002027 Epub 2011/05/19. 10.1371/journal.ppat.1002027 PPATHOGENS-D-10-00142 [pii]. 21589640 PMC3088726

[51] Serruto D, Spadafina T, Ciucchi L, Lewis LA, Ram S, Tontini M, et al *Neisseria meningitidis* GNA2132, a heparin-binding protein that induces protective immunity in humans. Proc Natl Acad Sci U S A. 2010;107(8):3770–5. 10.1073/pnas.0915162107 20133713 PMC2840514

