# Supplementary material for: The Phosphocarrier Protein HPr Contributes to Meningococcal Survival during Infection
Source: PLoS One. 2016 Sep 21;11(9):e0162434. doi: 10.1371/journal.pone.0162434 (PMC5031443; doi:10.1371/journal.pone.0162434)
Supplement: S1 Fig — (PDF) [file pone.0162434.s001.pdf]

**Fig. S1**

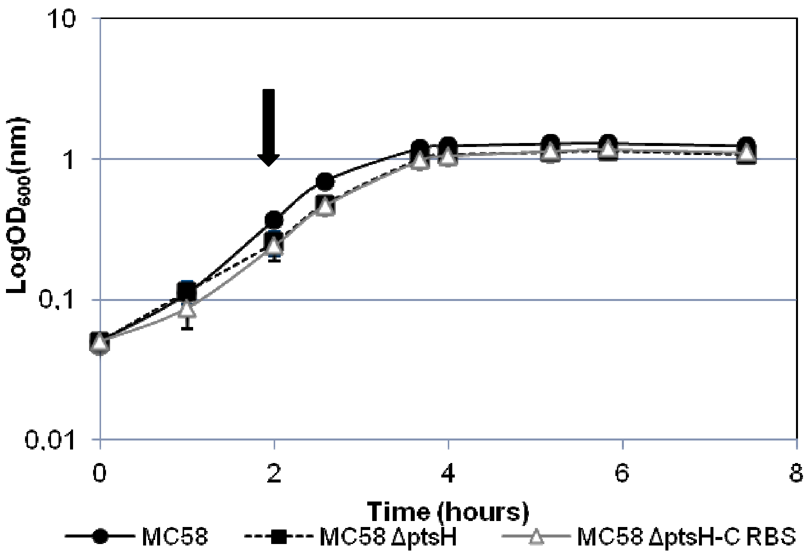


**Fig. S1. Growth curves from MC58, Δ*ptsH*, or Δ*ptsH*-C$_{RBS}$ strains in GCB media**. The arrow indicates the time point (mid-log) when RNA was extracted to perform transcriptional profile by microarrays analysis.
