# Supplementary material for: The Phosphocarrier Protein HPr Contributes to Meningococcal Survival during Infection
Source: PLoS One. 2016 Sep 21;11(9):e0162434. doi: 10.1371/journal.pone.0162434 (PMC5031443; doi:10.1371/journal.pone.0162434)
Supplement: S2 Fig — (PDF) [file pone.0162434.s002.pdf]

Fig. S2

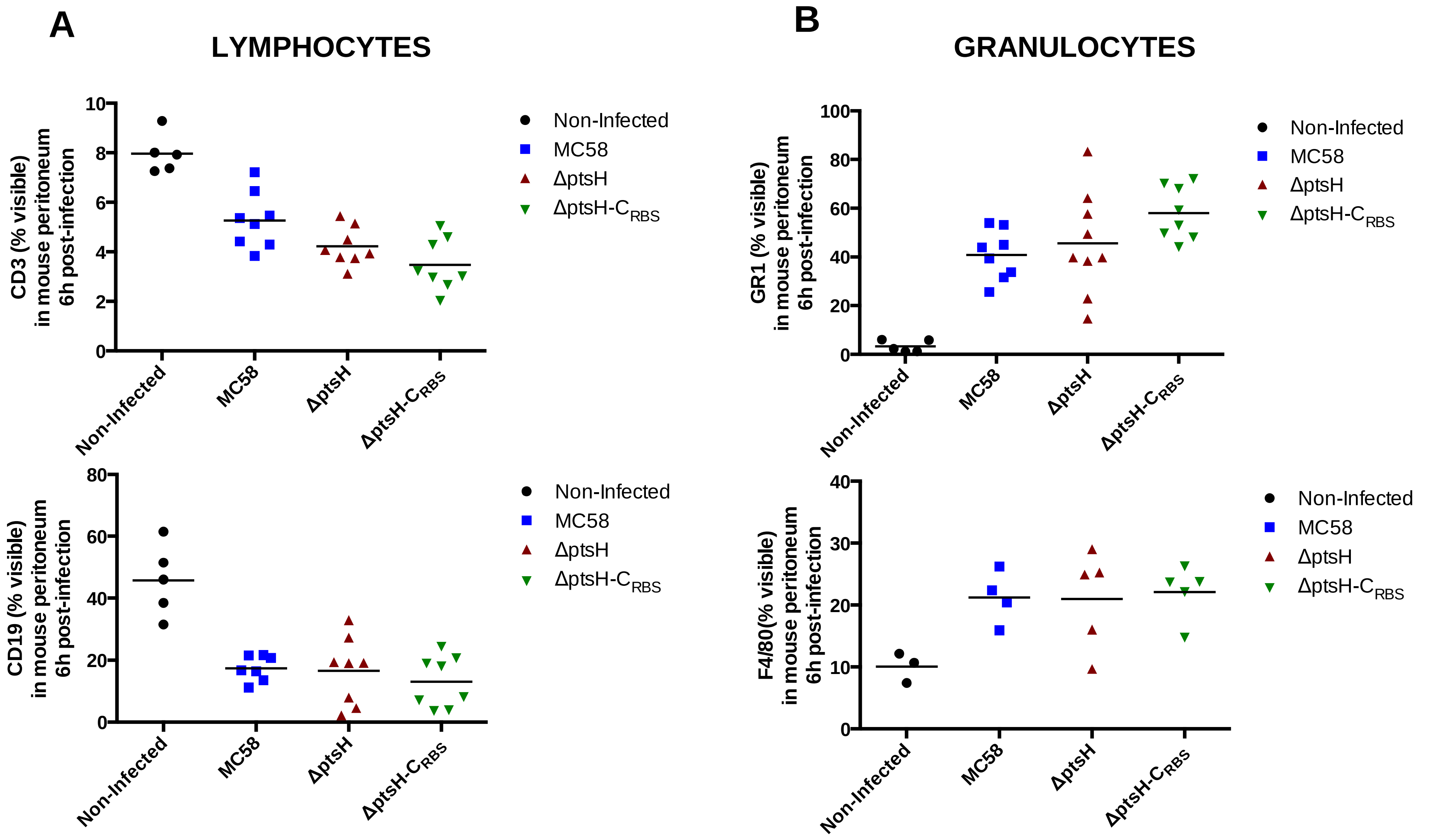

Fig. S2. Strain lacking HPr does not show different inflammatory cell recruitment by the host at the site of infection compared to wild-type and complemented strains. (A) Lymphocytes were analyzed by FACS using CD3 and CD19 markers from peritoneum washes after 6 h post-infection. (B) Granulocytes were analyzed by FACS using GR1 and F4/80 markers from peritoneum washes after 6 h post-infection.
