# Supplementary material for: The Phosphocarrier Protein HPr Contributes to Meningococcal Survival during Infection
Source: PLoS One. 2016 Sep 21;11(9):e0162434. doi: 10.1371/journal.pone.0162434 (PMC5031443; doi:10.1371/journal.pone.0162434)
Supplement: S3 Fig — (PDF) [file pone.0162434.s003.pdf]

Fig. S3

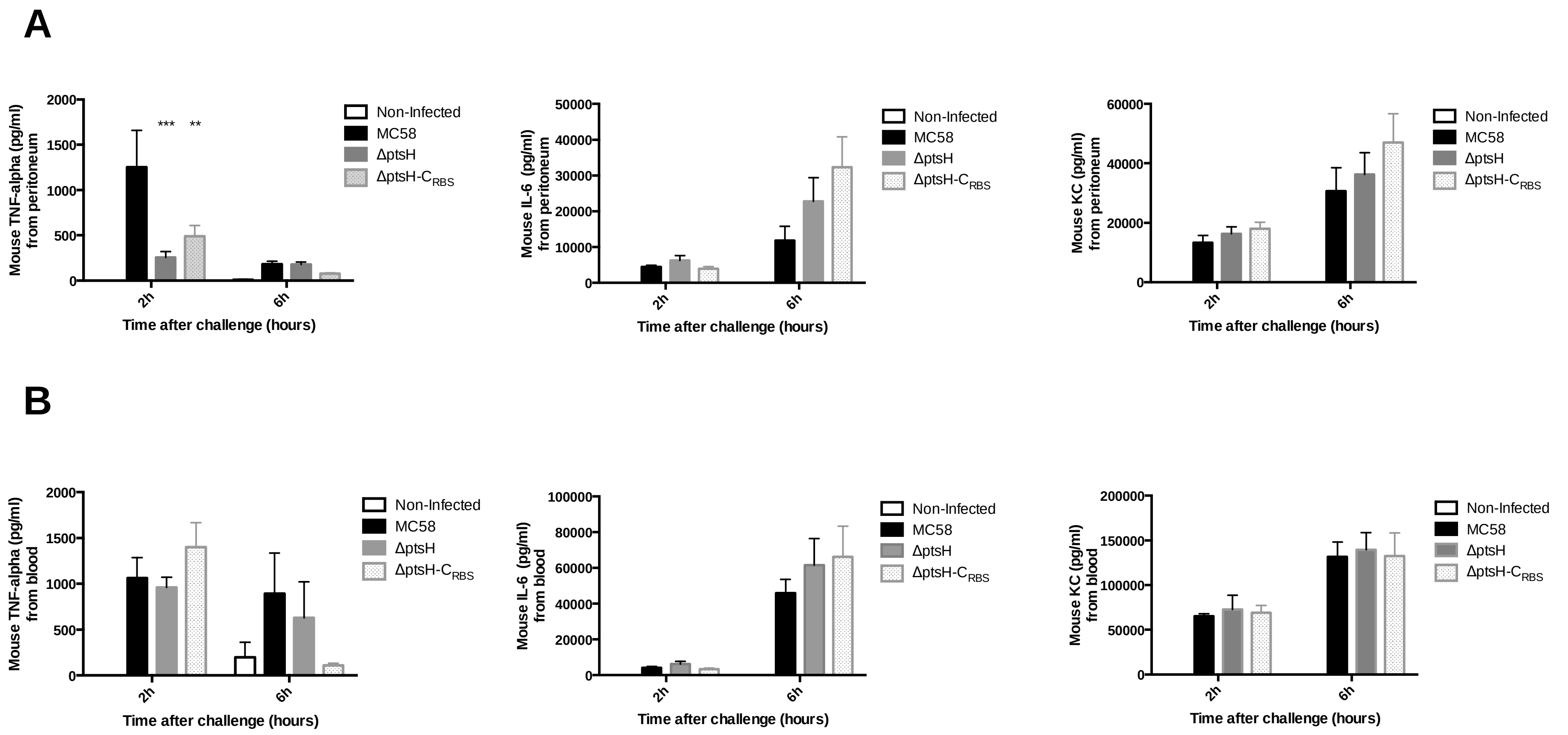

Fig. S3. Kinetics of cytokines production in BALB/c hTf transgenic mice infected by intra-peritoneal injection of 1 x 10^{7} CFU of MC58, Δ*ptsH*, or Δ*ptsH*-C$_{RBS}$ strains. ELISA assays were used to quantify TNF-alpha, IL-6 and KC from (**A**) peritoneum washes of the infection site (local infection) and from (**B**) blood samples (systemic infection) taken from mice after 2 and 6 h post-infection. Each bar represents the mean (with standard error of mean). The tested cytokines were not detectable in non-infected mice (not shown) and the comparisons were made for each anatomical site between samples from infected mice with MC58, Δ*ptsH*, or Δ*ptsH*-C$_{RBS.}$ Statistical analysis used two-tailed Mann-Whitney test with Bonnferoni correction for multiple comparisons. (*** $P < 0.001$ and ** $P < 0.01$ compared to the wild-type strain).
