# Supplementary material for: The Phosphocarrier Protein HPr Contributes to Meningococcal Survival during Infection
Source: PLoS One. 2016 Sep 21;11(9):e0162434. doi: 10.1371/journal.pone.0162434 (PMC5031443; doi:10.1371/journal.pone.0162434)
Supplement: S4 Fig — (PDF) [file pone.0162434.s004.pdf]

Fig. S4

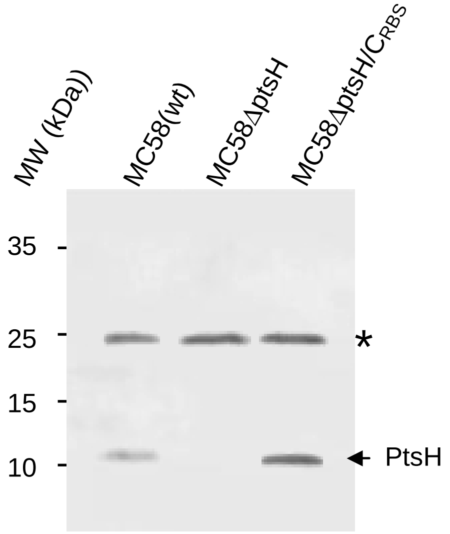

Fig S4. Immunoblot analysis of PtsH expression . Whole bacterial lystates derived from MC58 (wt), PtsH depleted mutant (MC58ΔptsH) and complemented strains were resolved in 14% SDS-PAGE and transferred to nitrocellulose membrane, then probed with a rabbit anti-PtsH directed serum. Molecular weigh (MW) markers are indicated in the left. The arrow indicates PtsH band. The asterisk correspond to a non-specific cross reactive band that allowed relative quantification of the PtsH
