# Supplementary material for: The Phosphocarrier Protein HPr Contributes to Meningococcal Survival during Infection
Source: PLoS One. 2016 Sep 21;11(9):e0162434. doi: 10.1371/journal.pone.0162434 (PMC5031443; doi:10.1371/journal.pone.0162434)
Supplement: S5 Fig — (PDF) [file pone.0162434.s005.pdf]

Fig. S5

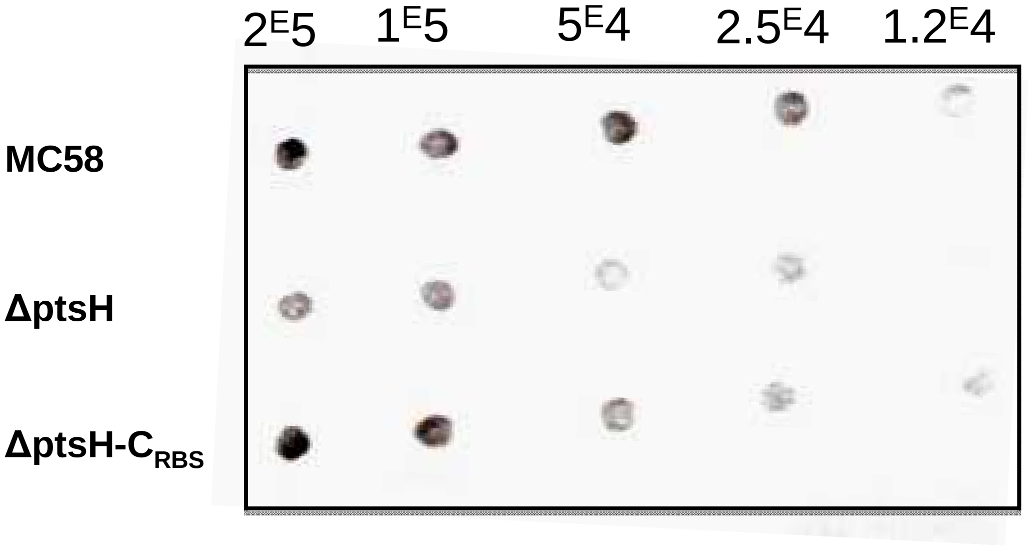

Fig S5. Dot blotting analysis for capsule quantification of MC58, Δ*ptsH*, and Δ*ptsH*-C$_{RBS}$ strains. Spots of 2 µl of bacterial suspensions of each indicated strains (right) were spotted onto nitrocellulose membrane. Colony forming units in each spot are indicated above. Antibodies used to detect capsular polysaccharide were the same as the ELISA.
