# Supplementary material for: The Phosphocarrier Protein HPr Contributes to Meningococcal Survival during Infection
Source: PLoS One. 2016 Sep 21;11(9):e0162434. doi: 10.1371/journal.pone.0162434 (PMC5031443; doi:10.1371/journal.pone.0162434)
Supplement: S6 Fig — (PDF) [file pone.0162434.s006.pdf]

Fig. S6

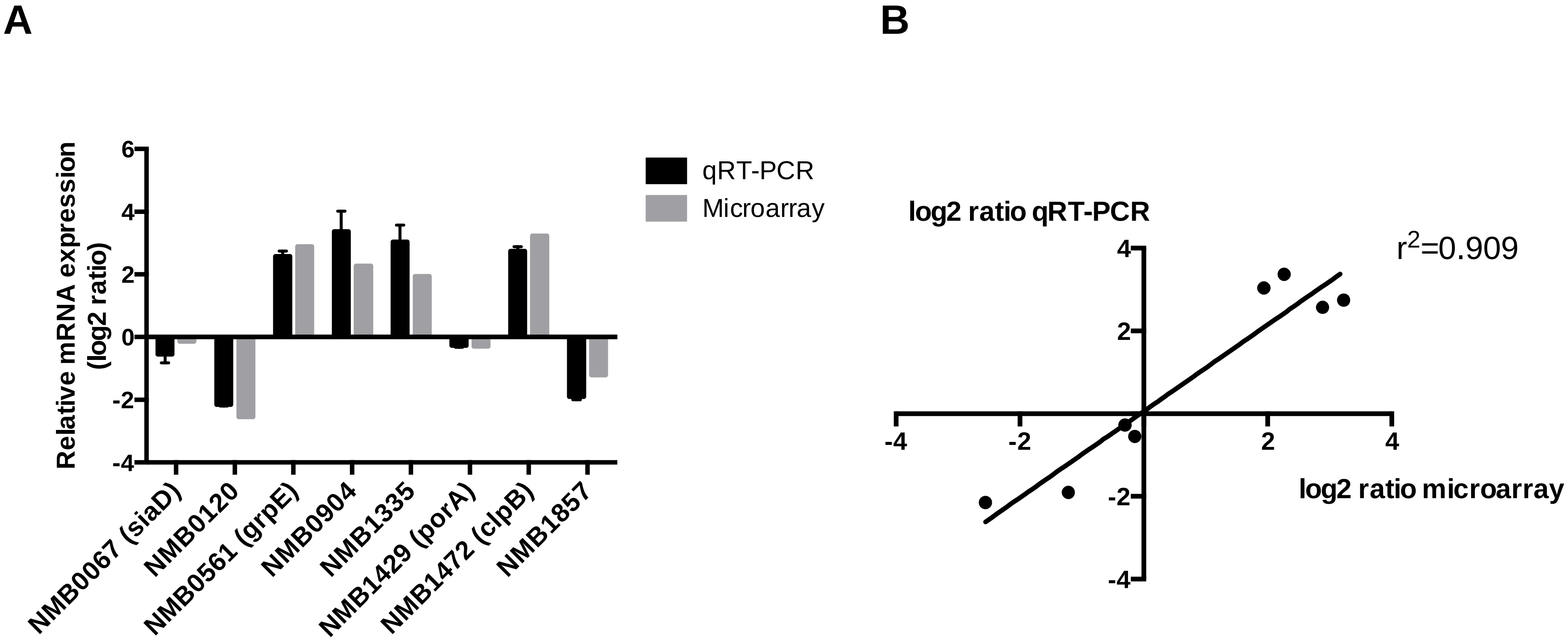

Fig. S6. Validation of microarray data obtained in this study. Solid bars indicate fold change values of selected genes, error bars indicate the standard deviation of three qRT-PCR biological replicates.(A) Comparison of microarray (grey bars) and qRT-PCR (black bars) expression data from 8 selected genes in Δ*ptsH* strain versus the MC58 strain when grown in GCB medium. (B) Correlation between microarray and qRT-PCR results for the 8 genes shown in (A).
