# Supplementary material for: The Phosphocarrier Protein HPr Contributes to Meningococcal Survival during Infection
Source: PLoS One. 2016 Sep 21;11(9):e0162434. doi: 10.1371/journal.pone.0162434 (PMC5031443; doi:10.1371/journal.pone.0162434)
Supplement: S7 Fig — (PDF) [file pone.0162434.s007.pdf]

Fig. S7

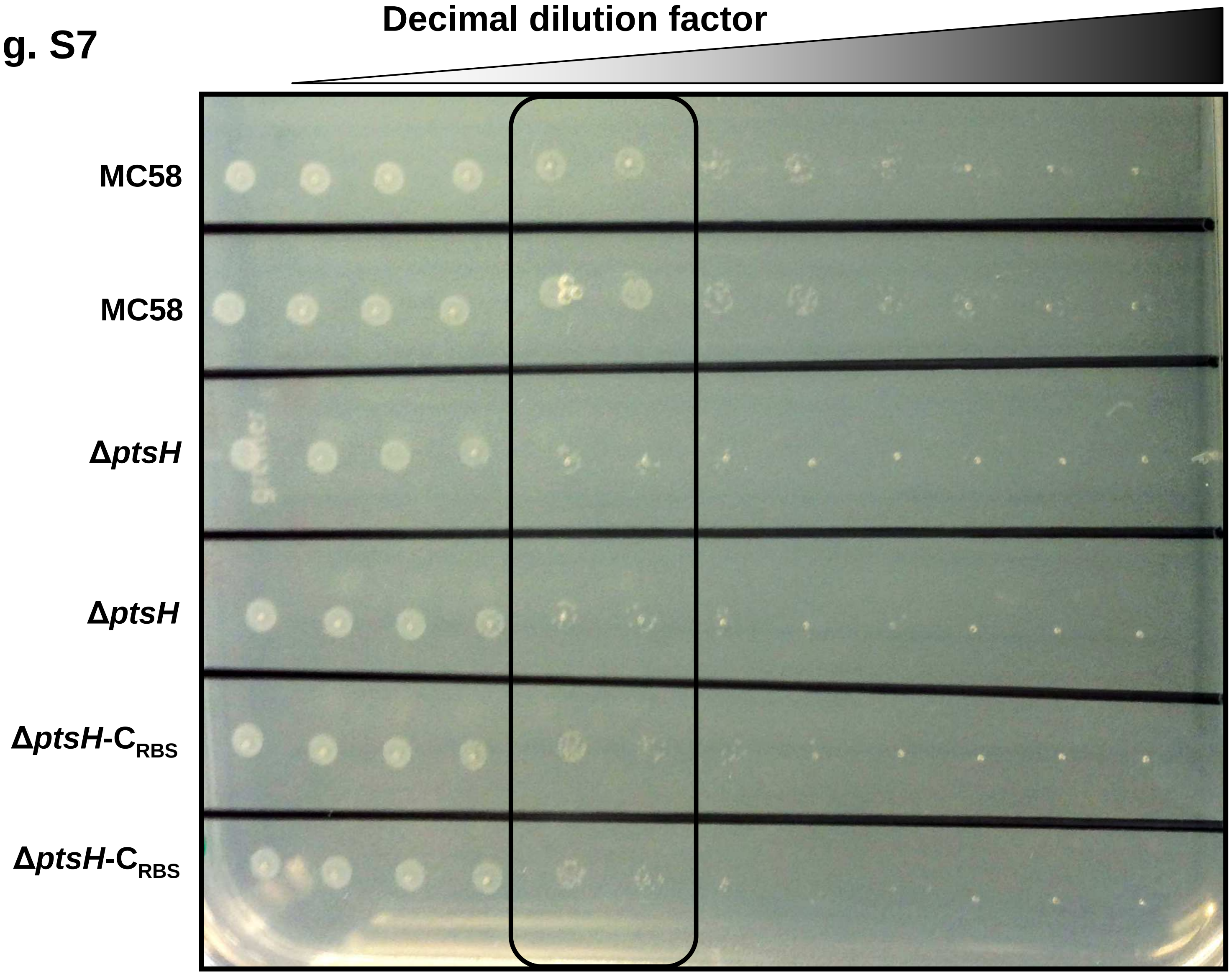

Fig. S7. HPr is important for resistance to oxygen depletion. Serial dilution plate assays were performed to compare the tolerance to lack of oxygen and the use of the nitrification pathway of MC58, Δ*ptsH*, Δ*ptsH*-C$_{RBS}$. Bacteria were grown in GCB with 10 mM $NaHCO_3$ + 5 mM $NaNO_2$ in an anaerobic jar. Image of a representative experiment.
